# Treatment Effects of Intra-Articular Allogenic Mesenchymal Stem Cell Secretome in an Equine Model of Joint Inflammation

**DOI:** 10.3389/fvets.2022.907616

**Published:** 2022-06-22

**Authors:** Clodagh M. Kearney, Sohrab Khatab, Gerben M. van Buul, Saskia G. M. Plomp, Nicoline M. Korthagen, Margot C. Labberté, Laurie R. Goodrich, John D. Kisiday, P. R. Van Weeren, Gerjo J. V. M. van Osch, Pieter A. J. Brama

**Affiliations:** ^1^School of Veterinary Medicine, University College Dublin, Dublin, Ireland; ^2^Department of Orthopaedics and Sports Medicine, Erasmus MC, University Medical Center Rotterdam, Rotterdam, Netherlands; ^3^Beacon Hospital, Dublin, Ireland; ^4^Department of Clinical Sciences, Faculty of Veterinary Medicine, Utrecht University, Utrecht, Netherlands; ^5^Equine Orthopaedic Research Center, Colorado State University, Fort Collins, CO, United States; ^6^Department of Otorhinolaryngology, Erasmus MC, University Medical Center Rotterdam, Rotterdam, Netherlands

**Keywords:** mesenchymal stem cells, secretome, joint inflammation, equine model, lipopolysaccharide (LPS)

## Abstract

**Background:**

Allogenic mesenchymal stem cell (MSC) secretome is a novel intra-articular therapeutic that has shown promise in *in vitro* and small animal models and warrants further investigation.

**Objectives:**

To investigate if intra-articular allogenic MSC-secretome has anti-inflammatory effects using an equine model of joint inflammation.

**Study Design:**

Randomized positively and negatively controlled experimental study.

**Method:**

In phase 1, joint inflammation was induced bilaterally in radiocarpal joints of eight horses by injecting 0.25 ng lipopolysaccharide (LPS). After 2 h, the secretome of INFy and TNFα stimulated allogeneic equine MSCs was injected in one randomly assigned joint, while the contralateral joint was injected with medium (negative control). Clinical parameters (composite welfare scores, joint effusion, joint circumference) were recorded, and synovial fluid samples were analyzed for biomarkers (total protein, WBCC; eicosanoid mediators, CCL2; TNFα; MMP; GAGs; C2C; CPII) at fixed post-injection hours (PIH 0, 8, 24, 72, and 168 h). The effects of time and treatment on clinical and synovial fluid parameters and the presence of time-treatment interactions were evaluated. For phase 2, allogeneic MSC-secretome vs. allogeneic equine MSCs (positive control) was tested using a similar methodology.

**Results:**

In phase 1, the joint circumference was significantly (*p* < 0.05) lower in the MSC-secretome treated group compared to the medium control group at PIH 24, and significantly higher peak synovial GAG values were noted at PIH 24 (*p* < 0.001). In phase 2, no significant differences were noted between the treatment effects of MSC-secretome and MSCs.

**Main Limitations:**

This study is a controlled experimental study and therefore cannot fully reflect natural joint disease. In phase 2, two therapeutics are directly compared and there is no negative control.

**Conclusions:**

In this model of joint inflammation, intra-articular MSC-secretome injection had some clinical anti-inflammatory effects. An effect on cartilage metabolism, evident as a rise in GAG levels was also noted, although it is unclear whether this could be considered a beneficial or detrimental effect. When directly comparing MSC-secretome to MSCs in this model results were comparable, indicating that MSC-secretome could be a viable off-the-shelf alternative to MSC treatment.

## Introduction

Osteoarthritis (OA) is a common debilitating disease in horses and humans (1). Given the fact that chronic and intermittent inflammation plays a predominant role in the prolonged disruption of joint homeostasis characteristic of OA, inflammation appears to be a logical target for novel therapeutics.

Mesenchymal stem cells (MSCs) are increasingly considered to be a promising biological treatment option for OA in horses and humans, and recently much focus has been on the use of allogenic MSCs (2). While there is still some discussion regarding the safety and efficacy of allogenic MSCs, more recent studies have shown that allogenic MSCs show similar effects to autologous MSCs in normal and inflamed joints (3), and a recent review concluded from accumulating evidence in studies to date in horses that allogenic MSCs are safe (2). Recently an allogenic mesenchymal stem cell product became the first stem cell-based veterinary medicine approved by the European Medicine Agency (4).

There is mounting evidence that the anti-inflammatory effects of MSCs result from their capacity to influence their micro-environment through the secretion of trophic factors (5–10). These secreted factors, known as secretomes are a cocktail of mediators and extracellular vesicles involved in many processes including inflammation and regeneration. Beneficial therapeutic effects of stem cell secretome were first described in the cardiovascular field, where a group investigating the potential therapeutic effects of MSCs on cardiomyocytes after exposure to hypoxia demonstrated *in vivo* that myocardial protection could also be afforded by concentrations of paracrine factors secreted by MSCs (11). The potential of these secreted factors to exert paracrine effects was naturally of interest in orthopedic research. While early experimental work with MSCs focused on exploring their capacity for differentiation and repair or regeneration of damaged joint tissues, the ability of MSCs to locally embed and replace damaged tissue is now known to be low (12, 13). Similar to the work with cardiomyocytes it has now been hypothesized that much of the therapeutic effectiveness of MSCS in joint disease is due to their release of paracrine factors which could counteract inflammatory and catabolic processes and foment endogenous repair (9, 14, 15). This has led researchers to investigate these secreted factors themselves as novel therapeutics rather than the parent MSCs. Our group and others have previously shown beneficial effects of MSC-secretome in *in vitro* and small animal *in vivo* OA models where an earlier reduction in pain and protective effects on cartilage were noted (15–17). If it would be possible to use the secretome as a therapeutic treatment instead of the cells themselves, it would provide opportunities to optimize the composition and concentration of these components *in vitro*. This would allow for an off-the-shelf cell-free treatment option with the potential to be widely available and affordable.

To the best of our knowledge, intra-articular administration of MSC-secretome has not previously been studied *in vivo* in the horse, although reports of its use in other areas have recently emerged (18). A research group from Cornell University has investigated various applications with regard to wound healing and found that conditioned medium from equine mesenchymal stem cells had both positive effects in an equine *in vitro* wound healing model (19) and also that equine MSC-secretome inhibits biofilm formation and mature biofilms of various bacteria (20). Lange-Consigilio et al. investigated conditioned medium from amniotic membrane-derived MSCs (AMC-CM) as an intralesional treatment in horses and ponies with naturally occurring tendon or ligament injuries and reported no adverse effects and favorable clinical outcomes (21). Those promising findings further supported our aim of investigating MSC-secretome in an equine model of joint disease.

In the presented study, we use a bilateral low dose LPS-induced inflammatory joint model in horses to first investigate the potential anti-inflammatory effects of allogenic MSC-secretome on clinical parameters and various biological markers in synovial fluid related to inflammation and cartilage turnover, compared to a control consisting of carrier medium only (negative control). Next, we compared the efficacy of intra-articular MSC-secretome to allogenic MSCs from the same cell lines the secretome was derived from (positive control). We hypothesized that intra-articularly injected MSC-secretome would demonstrate anti-inflammatory effects in this equine model of joint inflammation, and that intra-articularly injected MSC-secretome would be as effective as MSCs in reducing inflammation.

## Materials and Methods

### Study Design

**Figure 1:**
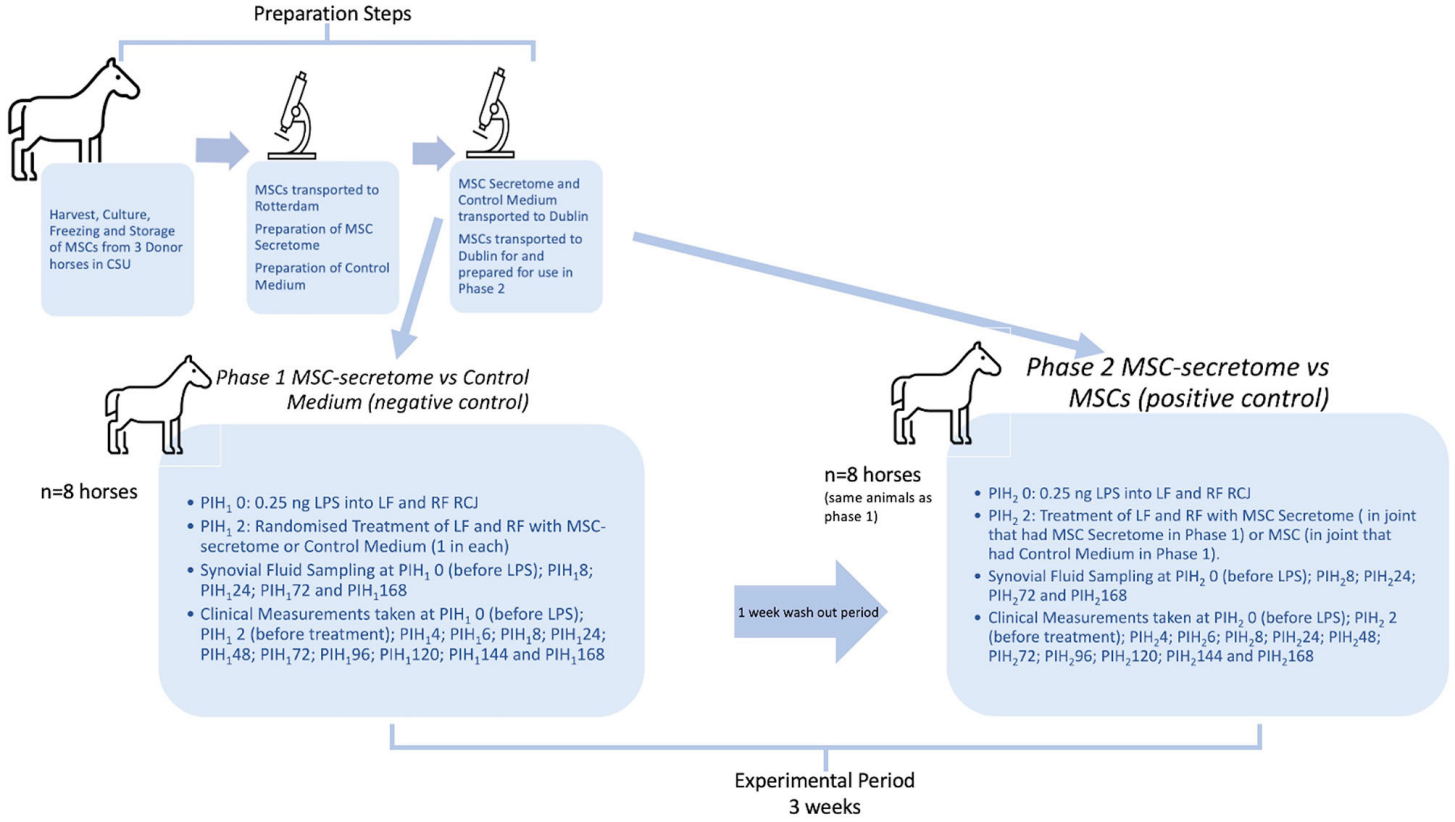
Overview of study design. The preparation steps were carried out in advance of the experimental period. Bone marrow derived mesenchymal stem cells (MSCs) were harvested from donor horses in Colorado State University (CSU) Veterinary Teaching Hospital and cultured, frozen and stored according to their standard protocols. Later, MSCs were transported, still frozen, to Erasmus MC in Rotterdam, where they were thawed and cultured and then used to prepare Mesenchymal stem cell secretome (MSC-secretome) treatments. Cells from the same cell lines as used for the MSC preparation were also transported to Dublin, where the final preparatory steps and viability assessment were performed immediately prior to their use in Phase 2 of the experiment. The experimental period represents 3 weeks in total. PIH (Post Induction Hour) indicates time in hours after induction of inflammation with intra-articular injection of 0.25 ng of lipopolysaccharide (LPS) in each radiocarpal joint (RCJ) of 8 horses. At PIH_1_2, one randomly selected RCJ of each horse was injected with intra-articular mesenchymal stem cell secretome and the contralateral joint was injected with medium (negative control). Following PIH_1_168, horses had a washout period (7 days) during which they were on pasture rest. At PIH_2_2, the RCJ that had previously been treated with intra-articular mesenchymal stem cell secretome was again treated with intra-articular mesenchymal stem cell secretome and the contralateral joint was injected with mesenchymal stem cells (positive control).

A complete overview of the study design is shown in Figure 1. In preparation for the experimental phase of the study, bone marrow-derived MSCs previously collected and stored at the Colorado State University Veterinary Teaching Hospital under the approval of the Institutional Animal Care and Use Committee of Colorado State University (15-5810A) were transported to the Erasmus Medical Center in Rotterdam. Using these cells MSC Secretome was prepared using techniques previously described for the production of secretome from Human bone marrow MSCs (17).Control medium was also prepared as a negative control, this product being the same formulation used to transport the MSC secretome but just not having been exposed to MSCs. Cells from the same cell lines as used for the MSC preparation were also transported to Dublin, where the final preparatory steps and viability assessment were performed immediately prior to their use in Phase 2 of the experiment.

For the experimental phase of the study 8 horses from the research herd of University College Dublin Lyons Research Farm were used following approval of the University College Dublin Animal Research Ethical Committee (AREC-16-29-Brama) and the Irish Health Products Regulatory Authority (AE18982-P105), in compliance with Irish legislation on experimental animal use. At the start of phase 1 both radiocarpal joints of each horse were injected with lipopolysaccharide (LPS) to induce joint inflammation. Two hours later one randomly selected radiocarpal joint of each horse was injected with intra-articular MSC secretome and the contralateral joint injected with control medium. Over the following week clinical parameters were measured and recorded, and serial synovial fluid samples were also taken during this period to determine the effect of each treatment on the joints involved. All investigators were unaware of the treatment assignment with the exception of the first author.

The same eight horses were used in both Phase 1 and Phase 2 of the study in an effort to reduce the numbers of experimental animals used so that each animal could act as its own control. Following a wash out period of 1 week after the last sampling, and 2 weeks after the first induction of inflammation with LPS Phase 2 of the study was initiated when inflammation was again induced in both radiocarpal joints of each horse with intra-articular injections of 0.25 ng of LPS. From previous work using the same dose of LPS intra-articularly, it was expected that all clinical and synovial markers of inflammation would be returned to baseline levels by this time (22). In this phase, the radiocarpal joint that had previously been treated with intra-articular MSC-secretome was again treated with intra-articular MSC-secretome and the contralateral joint was injected with mesenchymal stem cells. Clinical measurements and synovial fluid samples were taken as before. Specific detail regarding each step of the study is documented in the following sections.”

#### Collection and Expansion of MSCs

Equine bone marrow-derived MSCs from three donors were collected at the Colorado State University Veterinary Teaching Hospital. The procedure of harvesting and culturing MSCs is previously described (23). Specific characterization of these MSCs was not performed, however, previously published reports from this laboratory can give us some indication of the likely behavior of these cells. In respect of specific criteria set out in a recent position paper in this journal in this journal (24) these cells should demonstrate plastic adherence (23), chondrogenic and osteogenic potential (25–27), high CD 90, and low to negligible MHCII expression (26, 28). The MSCs were cryopreserved in a freeze media comprised of 95% fetal bovine serum (FBS) and 5% dimethyl sulfoxide (DMSO) and stored at −80°C prior to being shipped to Rotterdam. There the MSCs were cultured using previously described procedures (17). Briefly, MSCs were thawed, counted, and plated at 50,000 cells/cm^2^ and, after 24 h, the flasks were rinsed to remove the non-adherent cells. When 70% confluency was achieved, MSC were trypsinized [0.25% trypsin/ethylenediaminetetraacetic acid (EDTA) solution (Life Technologies)] and seeded in cell culturing flasks at a density of 2,300 cells/cm^2^ in expansion medium consisting of minimal essential medium alpha (αMEM; Gibco), 10% heat inactivated fetal calf serum (FCS; Gibco), 1.5 μg/ml fungizone (Invitrogen), 50 μg/ml gentamicin (Invitrogen), 25 μg/ml ascorbic acid-2-phosphate (Sigma-Aldrich) and 1 ng/mL fibroblast growth factor 2 (FGF2; AbD Serotec, Oxford, UK). Cells were cultured in an incubator at 37°C, 5% CO_2_, and 90% humidity. The medium was refreshed 2 times a week. MSCs were passaged at ~70% confluency. The cells were passaged three times in a monolayer prior to being used in the experimental protocols.

#### Preparation of MSC-Secretome and Control Medium

The dose of secretome per joint was planned to be the secretome equivalent of 10 × 10^6^ MSCs. To produce the MSC-secretome, passage 3 MSCs were plated at a density of 3.5 × 10^4^ cells/cm^2^ and cultured for 24 h in an expansion medium. After 24 h, cells were activated to secrete immunomodulatory factors by culturing for 24 h in stimulating medium (15, 17). This stimulating medium consisted of αMEM supplemented with 1.5 μg/ml fungizone, 50 μg/ml gentamicin, 1% insulin–transferrin–selenium (ITS; Biosciences), 50 ng/ml equine interferon gamma (Recombinant Equine IFN-gamma Protein, R&D) and 50 ng/ml equine tumor necrosis factor alpha (Recombinant Equine TNF-alpha Protein, R&D). After 24 h of stimulation, MSCs were washed five times with phosphate-buffered saline (PBS; Gibco). To collect the paracrine factors, a collecting medium was added, consisting of only αMEM (MEM α, nucleosides, no phenol red, ThermoFisher) with 0.05% equine serum albumin (ESA; Rocky Mountain Biologicals Inc.)—to stabilize the secreted factors and as an adhesive for smaller molecules to bind to and to be retained after the concentration step—and without phenol red that can mimic estrogen and therefore influence cell behavior *in vivo*. About 1 ml of collecting medium was added per 2.0 × 10^5^ MSCs. MSC-secretome was collected after 24 h and centrifuged at 700 × g for 8 min to remove cell debris. To achieve the desired concentration (secretome equivalent of 10 × 10^6^ MSCs) in an end volume of 3 ml, suitable for intra-articular injection, the MSC-secretome was concentrated, according to a previously developed protocol by our lab (17). Briefly, this was done by loading MSC-secretome on a 3 kDa cut-off filter (Merck Millipore Centricon Plus-70 device, 3K) and spinning down for 20 min at 4,000 ×g. Molecules above 3 kDa were retained. The concentrated equine MSC-secretome was collected, aliquoted, and stored at −80°C for further use. For each injection concentrated MSC-secretome from each of the three donors was pooled to give aliquots of a final volume of 3 ml, representing the secretome of 10 × 10^6^ MSC.

Control medium was prepared by subjecting the collecting medium used for the MSC-Secretome—αMEM (with no phenol red) and 0.05% equine serum albumin—to the same handling as the MSC-secretome, including 24 h incubation and concentration step, but not including exposure to the MSCs, and then stored at −80°C until required.

Both the MSC-secretome and the control medium were thawed on ice immediately prior to injection.

#### Preparation of MSC Injections

Circa 24 h prior to injection the culture flasks containing MSCs from the same cell lines as used for the production of MSC-secretome, were washed five times with phosphate-buffered saline (PBS; Gibco). Whereafter the same collecting medium as in the MSC-secretome preparation was added, consisting of only αMEM w/o phenol red with 0.05% equine serum albumin (ESA; Rocky Mountain Biologicals Inc.). Unlike the cells used for the MSC-secretome production, these MSCs were not stimulated with equine interferon gamma and equine tumor necrosis factor alpha as it was considered they would be exposed to an inflammatory environment in the LPS-inflamed joints. After 24 h, the MSCs were trypsinized and the MSCs were collected. The viability of the MSCs was evaluated after trypsinization: <5% of the cells were dead, as indicated by visual assessment following trypan blue positive staining. For each intra-articular injection, cells were pooled from each donor to give a total of 10 ×10^6^ MSC collected in a volume of 3 ml of control medium. The cells were injected within 2–4 h of trypsinization and evaluation.

### Experimental Animals

Eight horses (16 joints) were selected to participate in a randomized controlled experiment. The animals of various breeds (six mares and two geldings) (mean ± SD age 14.6 ± 2.4 years, bodyweight 370.4 ± 27.6 kg) were from the University research herd. There was no known history of forelimb lameness in any of the animals. Each animal was examined clinically by 2 ECVS boarded surgeons, and was found to have no sign of forelimb lameness. On clinical and radiographic examinations their carpal joints were found to be within normal limits. While individual animals were previously used in other experimental studies the radiocarpal joints of these animals had not previously been injected or treated in any way. During the sampling phases of the experiment, the animals were stabled individually in single boxes (4 m × 4 m) on wood shavings. Horses received concentrates once daily, with regular hay and water provided *ad libitum*. Following the week of sampling and measurements during which the horses were stabled, they were then turned out to pasture in a familiar group for a week. They were brought back in on the morning of the second induction of LPS and were again stabled under the same conditions during this second week of sampling and measurements. Before commencement of Phase 2 of the study, each animal was again examined by two ECVS boarded surgeons and was found to be free of any forelimb lameness and of any clinical signs of inflammation of the radiocarpal joints (joint effusion, heat, or pain on palpation or flexion).

### Experimental Protocol

#### Induction of Inflammation

At post induction time (PIH) 0, both carpi of each horse were clipped and prepared for dorsal arthrocentesis. Lipopolysaccharide from *Escherichia coli* O55:B5 (catalog number L5418; Sigma-Aldrich Ireland Ltd., Arklow, Co. Wicklow Ireland) was diluted to a final concentration of 0.25 ng/ml in sterile lactated Ringer's solution. Horses were sedated with xylazine (0.2–0.5 mg/kg intravenously, Chanazine 10%^®^ Chanelle, Ireland) and butorphanol (0.01–0.02 mg/kg intravenously; Alvegesic vet 10^®^, ALVETRA u. WERFFT GmbH, Vienna, Austria). Synoviocentesis was performed in each limb with a 20 G × 40 mm needle and 1 ml LPS solution (0.25 ng LPS) was delivered aseptically into each radiocarpal joint after withdrawal of the PIH 0 synovial fluid (SF) sample.

#### Treatments

##### Phase 1 MSC-Secretome vs. Medium (Negative Control)

In the first phase of the experiment, 2 h following induction of inflammation with LPS (PIH_1_2), following preparation of the regions as before, one randomly assigned radiocarpal joint of each horse was injected with 3 ml of allogeneic MSC-secretome (treatment), and the opposite radiocarpal joint was injected with the same volume of control medium (negative control).

##### Phase 2 MSC-Secretome vs. MSCs (Positive Control)

Following a wash-out period of 1 week after the last sampling, and 2 weeks after the first induction of inflammation with LPS, the same group of horses was used for Phase 2 of the study. From previous work using the same dose of LPS intra-articularly, it was expected that all clinical and synovial markers of inflammation would be returned to baseline levels by this time (22). In this second phase of the experiment, 2 h following induction of inflammation with LPS (PIH_2_2), following preparation of the regions as before, the same radiocarpal joint as had been treated with allogeneic MSC-secretome in the previous phase was injected with secretome (treatment), and the opposite radiocarpal joint was injected with allogeneic MSCs (positive control).

### Clinical Evaluations

#### Welfare Monitoring

Before synoviocentesis and induction of inflammation and again every 2 h until PIH 8, and thereafter daily until PIH 168 a Composite Welfare Score (CWS) was assigned by an experienced vet. The CWS is the sum of scores for each of the following categories: food and water intake; clinical parameters (temperature, pulse, and respiratory rate); natural behavior; and provoked behavior. Each of the categories is scored on a scale of 0–4, so the total range of scores is 0–16. This scoring system has been designed by our group for this bilateral equine LPS model to monitor welfare and to fulfill institutional and national ethical regulatory requirements (scoresheet available in supporting information).

#### Clinical Measurements

In each induction, before synoviocentesis at PIH 0, every 2 h until PIH 8, and thereafter daily until PIH 168, radiocarpal joint effusion was graded on a subjective scale as previously described (29). An experienced clinician carefully palpated the joints and assigned a score ranging from 0 to 4; a score of 1, 2, or 3 denoting mild, moderate, or severe radiocarpal joint effusion, respectively, and 4 indicating severe swelling of the entire carpal region. In addition, joint circumference was measured at a fixed anatomical landmark at the level of the accessory carpal bone with a tape measure in mm. At the start of each phase, a mark was drawn on the skin over the accessory carpal bone to use as a reference point so that all measurements would be taken at the same level. All clinical measurements were performed by the first author and therefore cannot be considered to be blinded.

### Synovial Fluid Analysis

At fixed time points (PIH 0, 8, 24, 72, and 168), synoviocentesis of each radiocarpal joint was performed under sedation as described above and a 4–5 ml sample of synovial fluid was collected. About 1.3 ml of this synovial fluid was placed in ethylenediamine tetra-acetic acid (EDTA) for manual white blood cell count (WBC) and total protein (TP) measurement (refractometer). The remainder was immediately centrifuged in plain tubes for 15 min at 4°C at 10,000 rpm and then aliquoted and stored at −80°C until further analysis.

#### Synovial Fluid Molecular Biomarker Analysis

Seven assays were performed on each synovial fluid sample.

Eicosanoid inflammatory mediators—Prostaglandin F2α (PGF2α), Prostaglandin E_2_ (PGE_2_), Prostaglandin E_1_ (PGE_1_), Leukotriene B_4_ (LTB_4)_, and 11-hydroxyeicosatetraenoic acid (11-HETE)—concentrations were determined by high-performance liquid chromatography (HPLC)–tandem mass spectrometry (MS/MS) analysis using previously validated methods (30). Briefly, measurements were made using a 4000 Q TRAP mass spectrometer with electrospray ionization (EPI) interface (Sciex, Toronto, ON), operated in multiple-reaction monitoring (MRM) mode at unit mass resolution. The mobile phases consisted of 10 mM ammonium acetate pH 3.5 in water, and 10 mM ammonium acetate pH 3.5 in methanol. Peaks were identified by comparison of retention time and mass spectra of standards using Analyst software version 1.6.2 (Applied Biosystems, Nieuwerkerk a/d IJssel, The Netherlands).

General matrix metalloproteinase (MMP) activity was measured using cleavage of fluorogenic substrate FS-6i (Calbiochem, San Diego, CA, USA) as previously described (31, 32). Briefly, samples were first diluted 20-fold in MMP buffer [0.1 mol/L Tris, 0.1 mol/l NaCl, 10 mmol/L CaCl_2_, 0.05% (w/v) Triton X-100, 0.1% (w/v) PEG6000, pH 7.5 and 5 mmol/L FS-6]. Samples were subsequently added in triplicate to a black 384-well microplate and the fluorescent signal was monitored continuously for 45 min at 37°C using a CLARIOstar microplate reader. The slope of the resultant linear curve [relative fluorescence units/s (RFU/s)] was then calculated as a measure of general MMP activity. A quantity of 5 mmol/L EDTA was used as a negative control.

Synovial fluid samples were evaluated for glycosaminoglycan (GAG) concentrations using a modified 1,9-dimethylmethyleneblue assay adapted for use in microtitre plates, as previously described (33).

C–C motif chemokine ligand 2 (CCL2) and tumor necrosis factor-α (TNF-α) concentrations were quantified using commercial equine-specific ELISA kits (DIY0694E-003 Kingfisher Biotech, Minnesota USA and #ESS0017, Thermo Fisher Scientific, Massachusetts, USA) using an adapted protocol as previously described (22). The coating buffer consisted of carbonate/bicarbonate buffer (pH 9.6) and the blocking/dilution buffer was PBS with 1% w/w bovine serum albumin (BSA) (Sigma Aldrich, Saint Louis, USA). Samples were diluted 1:1 in PBS/1% BSA/0.1% (v/v) Tween-20, and results were calculated to a standard curve plotted on four parameters logistic curve fit. Values equal to, or below the blank were set to zero.

Commercial ELISA kits were used to determine concentrations of collagen-cleavage neoepitope of type II collagen (C2C), and carboxypropeptide of type II collagen epitope (CPII) (IBEX Technologies, Quebec, Canada), following the manufacturer's recommendations. Samples for C2C were 1:1 diluted and for CPII were 1:10 diluted, both in buffer III, and results were calculated to a standard curve plotted on four parameter logistic curve fit. Values equal to, or below the blank were set to zero.

GAGs, CCL2, TNF-α, C2C, CPII, GAG were all quantified on a VersaMax™ ELISA microplate reader. GAGs were measured at 525 and 595 nm and all the ELISAs were measured according to the manufacturer's recommendations.

### Statistical Analysis

An a priori power analysis was performed. The power calculation was based on previous similar studies using the LPS model with described differences in synovial fluid biomarkers indicating joint inflammation (31, 34, 35). The power calculation suggested that eight horses would give a power of 0.8 and an alpha error rate of 0.05. Data are presented as the mean ± standard deviation (SD).

For each phase, a linear mixed effects model for repeated measures was fitted, with the horse as a random effect and time, treatment, and their interaction as fixed effects. An Independent variance-covariance structure was used in the model. Planned univariate contrasts (Wald tests) were performed between marker concentrations in MSC-secretome (treatment) and medium (negative control) (Phase 1), or MSC-secretome (treatment) and MSC (positive control) (Phase 2) treated joints at specific time points following observation of an overall significant effect of treatment, using Bonferroni's correction for multiple comparisons, with each phase considered as a separate experiment. Normality was assessed by visual inspection of plots of standardized residuals. The suitability of the mixed effects model over a linear model was assessed by AIC, BIC, and Likelihood Ratio Test. Computer software was used (*Stata Statistical Software: Release 15*. StataCorp LLC, College Station, TX) and the level of significance was set at *p* < 0.05 for all statistical analyses (*p* < 0.025 with Bonferroni correction).

## Results

### Phase 1: MSC-Secretome (Treatment) vs. Medium (Negative Control)

#### Validation of Inflammatory Response

**Figure 2:**
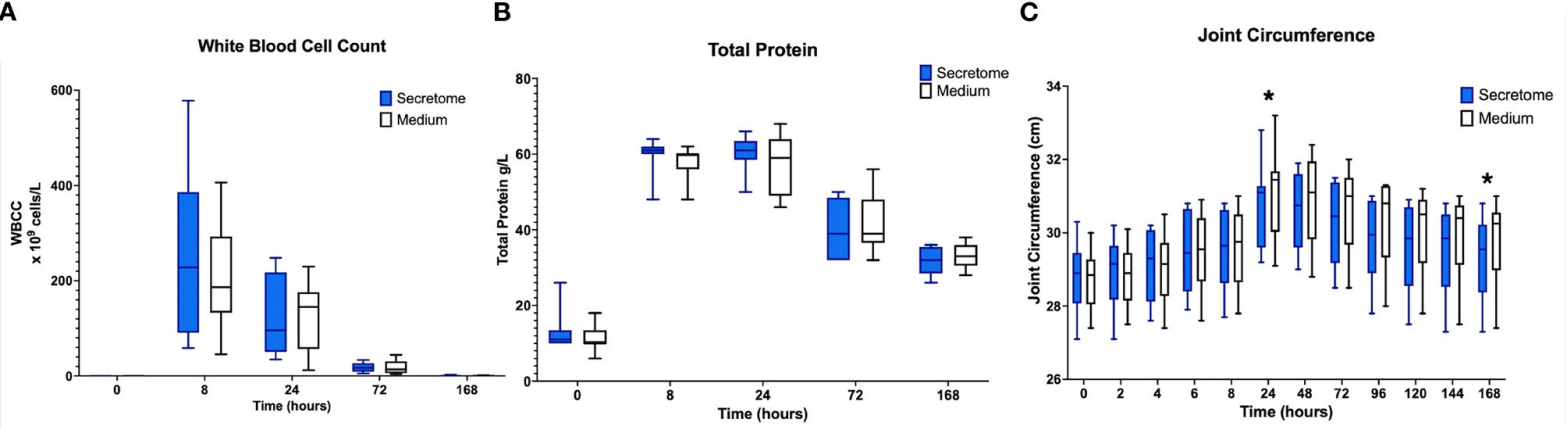
Phase 1 synovial white blood cell counts, total protein, and joint circumference. **(A)** Synovial White Blood Cell Count, **(B)** Synovial Fluid Total Protein, and **(C)** Joint Circumference over time following induction of inflammation with intra-articular injection of 0.25 ng of LPS in the left and right radiocarpal joints of horses at PIH_1_ 0 (*n* = 8 horses). Joints were treated with either intra-articular mesenchymal stem cell (MSC) secretome or medium (negative control) at PIH_1_2. Boxes depict median and interquartile ranges; whiskers denote minimum and maximum values. For the Synovial White Blood Cell Count, the box and whiskers for the first timepoint are not visible on the graph as the values for these were very low with each measurement recorded being < 1 × 10^9^ cells/L. *p < 0.05, indicating time points where there are significant treatment effects.

In both control and treated limbs, clear inflammatory responses, in the form of the expected peaks and subsequent falls in total protein and synovial white blood cell counts were seen after administration of LPS (Figures 2A,B).

#### Welfare Monitoring

For those horses that had slight Composite Welfare Score (CWS) increases in the early stages of the period of inflammation, their scores had returned to the normal range by 24 h post induction (Supplementary Table S2).

#### Clinical Monitoring

For the primary research question investigating the effects of intra-articular administration of secretome on joint circumference a statistically significant treatment effect was seen with a reduction in joint circumference in the MSC-secretome treated group compared to the control treated group at PIH 24 (−0.33125 cm, *p* = 0.0247) and at PIH 168 (−0.45 cm, *p* = 0.0012) (Figure 2C). From the data in Supplementary Table S3 it appears that joint circumference in both treatment groups remains above baseline levels at PIH 168, although it is not known whether these are significant differences as contrasts comparing each timepoint in each treatment group to baseline values were not performed. As joint effusion scores were on an ordinal scale, after consideration of the repeated measures design, in particular in conjunction with the small sample size (*n* = 8), formal statistical methods such as ordinal logistic regression were considered inappropriate. No appreciable differences were apparent from simple observation between treatment groups. Results are summarized in Supplementary Table S3.

#### Synovial Fluid Molecular Biomarker Monitoring

The results for all synovial fluid parameters are summarized in Supplementary Table S4, which also includes where available our laboratory's baseline ranges for each synovial fluid biomarker.

**Figure 3:**
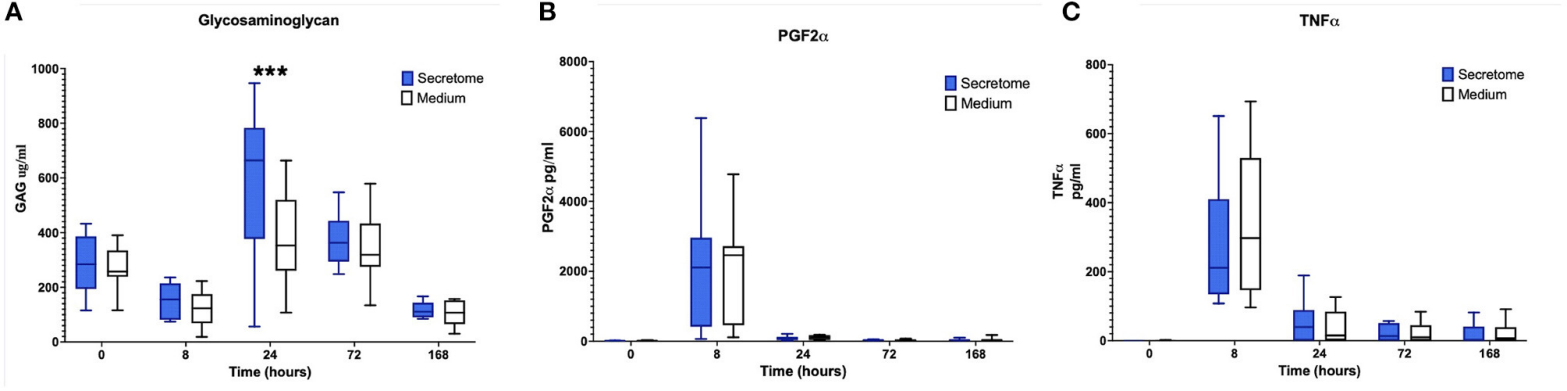
Phase 1 synovial fluid glycosaminoglycan, prostaglandin F2α and tumor necrosis factor α. **(A)** Glycosaminoglycan, **(B)** Prostaglandin F2α, and **(C)** Tumor Necrosis Factor α concentrations in synovial fluid over time following induction of inflammation with intra-articular injection of 0.25 ng of LPS in the left and right radiocarpal joints of horses at PIH_1_ 0 (*n* = 8 horses). Joints were treated with either intra-articular mesenchymal stem cell (MSC)-secretome or medium (negative control) at PIH_1_2. Boxes depict median and interquartile ranges; whiskers denote minimum and maximum values. ***p < 0.001, indicating timepoints where there are significant treatment effects.

Regarding the effects of intra-articular administration of secretome on synovial concentrations of biomarkers, results indicate a difference in treatment effect with increases in GAG concentrations in the MSC-secretome treated group compared to the control treated group in the first phase at PIH 24 (+201.29 μ/ml, *p* = 0.00067) (Figure 3A). For the other biomarkers, treatment effects are not evident, as illustrated for selected markers in Figures 3B,C.

Summarizing the results of the comparison between MSC-secretome and medium indicated that MSC-secretome reduces joint circumference and influences GAG release, but not other synovial fluid cartilage turnover or inflammation markers.

### Phase 2: MSC-Secretome (Treatment) vs. MSCs (Positive Control)

#### Validation of Inflammatory Response

**Figure 4:**
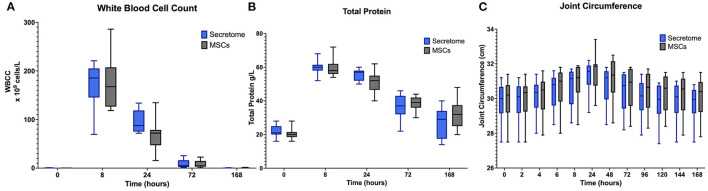
Phase 2 synovial white blood cell counts, total protein and joint circumference. **(A)** Synovial White Blood Cell Count, **(B)** Synovial Fluid Total Protein, and **(C)** Joint Circumference over time following induction of inflammation with intra-articular injection of 0.25 ng of LPS in the left and right radiocarpal joints of horses at PIH_2_ 0 (*n* = 8 horses). Joints were treated with either intra-articular mesenchymal stem cell (MSC)-secretome of mesenchymal stem cells (MSCs) (positive control) at PIH_2_2. Boxes depict median and interquartile ranges; whiskers denote minimum and maximum values. For the Synovial White Blood Cell Count the box and whiskers for the first timepoint are not visible on the graph as the values for these were very low with each measurement recorded is < 1 × 10^9^ cells/L.

In both groups (MSC and MSC-secretome treated joints) clear inflammatory responses in the form of the expected peaks and subsequent falls in synovial white blood cell counts and total protein were seen after administration of LPS (Figures 4A,B).

#### Welfare Monitoring

As in Phase 1 for horses that had slight CWS increases in the early stages of the period of inflammation, their scores had returned to the normal range by 24 h post induction (Supplementary Table S2).

#### Clinical Monitoring

A potentially confounding finding was that from Supplementary Tables S3, S5 it can be seen that for both treatment groups the joint circumference was slightly higher at Timepoint 0 of Phase 2 than at Timepoint 168 of Phase 1. This was unexpected as the measurements had been decreasing toward the end of Phase 1 and the horses were carefully checked at the start of Phase 2 and no evidence of joint effusion was recorded at Timepoint 0. This apparent discrepancy would seem to be due to some inconsistency in the placement of the marks drawn on the skin over the accessory carpal bone meaning that measurements were taken at slightly different levels between groups.

For joint circumference, while from PIH 24 onwards the values of the MSC-secretome treated group appeared lower than those of the MSC treated group these differences were not found to be significant (Figure 4C). For joint effusion scores, as in Phase 1, no appreciable differences were observed between treatment groups. Results are summarized in Supplementary Table S5.

#### Synovial Fluid Molecular Biomarker Monitoring

The results for all synovial fluid parameters are summarized in Supplementary Table S6.

**Figure 5:**
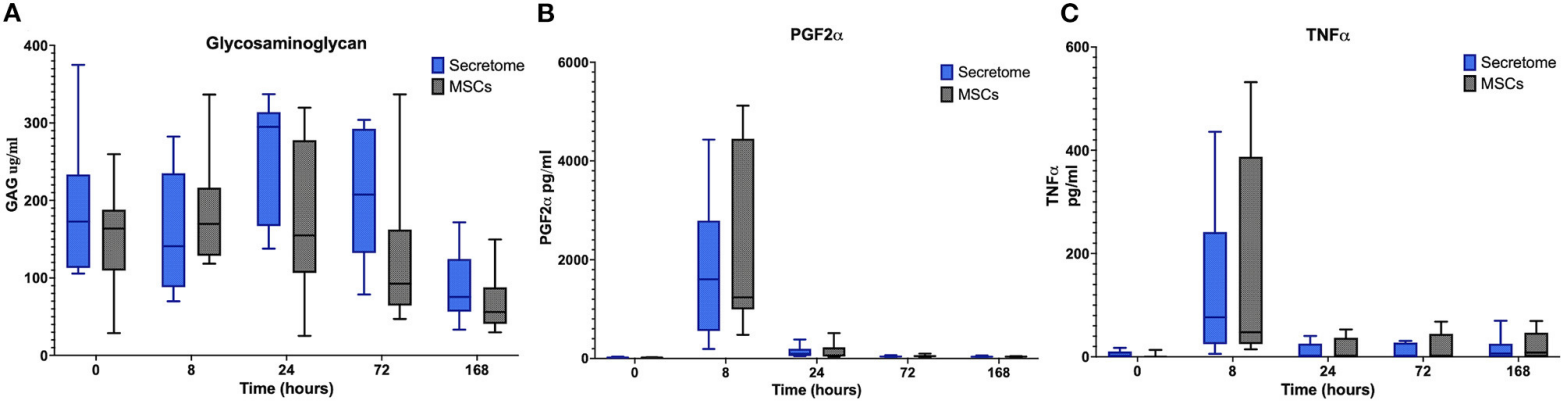
Phase 2 synovial fluid glycosaminoglycan, prostaglandin f2α and tumor necrosis factor α. **(A)** Glycosaminoglycan, **(B)** Prostaglandin F2α, and **(C)** Tumor Necrosis Factor α concentrations in synovial fluid over time following induction of inflammation with intra-articular injection of 0.25 ng of LPS in the left and right radiocarpal joints of horses at PIH_2_ 0 (*n* = 8 horses). Joints were treated with either intra-articular mesenchymal stem cell (MSC)-secretome of mesenchymal stem cells (MSCs) (positive control) at PIH_2_2. Boxes depict median and interquartile ranges; whiskers denote minimum and maximum values.

No significant differences between the MSC-secretome treated and MSC treated joints were noted for any clinical or synovial fluid biomarker as illustrated for selected markers in Figure 5. For synovial GAG, the peak value of the MSC-secretome treated group was higher than the peak value of the MSC treated group at PIH 24 but this did not reach significance (*p* = 0.029) (Figure 5B).

In summary, the comparison between MSC-secretome and MSCs revealed no significant difference in treatment effect.

## Discussion

In this study, we compared the effect of intra-articular allogenic MSC-secretome in an equine within-animal-controlled model of joint inflammation to negative control (medium) and positive control (allogenic MSCs). We report two main findings. First, when compared to negative control, intra-articular allogenic MSC-secretome reduces joint circumference and increases GAG release at the 24-h timepoint (PIH 24) in an equine model of LPS induced synovial inflammation. Second, when compared in the same equine LPS model of synovial inflammation, no significant differences in treatment effects of intra-articular allogenic MSC-secretome vs. allogeneic MSCs were detected.

In our previous *in vivo* study assessing the effects of MSC-secretome injection in a murine OA model, clinical benefits such as an early reduction in pain as determined by increased weight bearing were seen (17). In the present study, clinical benefit seen as a significant reduction in carpal circumference in the group of horses treated with MSC-secretome was noted, corroborating what was found in the earlier mouse model.

The previous *in vitro* work also demonstrated anti-inflammatory and matrix turnover altering effects of MSC secretome on human osteoarthritic cartilage and synovium (11). In addition, we found a reduction in cartilage damage after MSC-secretome injection in our murine OA model study (15, 17). Other groups have shown protective effects of MSC-secretome in an inflammatory *in vitro* chondrocyte model (16) and beneficial effects of MSC-derived extracellular vesicles in various pre-clinical OA models *in vivo* (36, 37). In the present study, we demonstrated a significant increase in levels of GAGs in the synovial fluid of secretome-treated joints compared to the control (medium treated) joints. In previous studies using GAG levels as outcome assessments when investigating intra-articular therapeutics increases in GAG levels (38, 39) have been varyingly explained as either a catabolic response due to an increased breakdown of GAGs already present in the cartilage, or as an anabolic response reflected by an increase in GAG production of the cartilage being exposed to an inflammatory environment. From our results, we cannot definitively assess whether the increased GAG concentration found in secretome treated joints was caused by a catabolic or an anabolic response, but the inclusion of further biomarkers such as the CS 846 epitope which has been found to be useful as a marker of aggregran synthesis (40) could help to clarify this in future studies.

MSCs have been studied as a potential form of cell therapy for equine joint disease in both experimental and clinical settings (41–44). Currently, in Europe, there are two approved veterinary stem cell-based products, namely allogenic blood or umbilical cord-derived mesenchymal stem cells, which lend credibility to their therapeutic potential. For this study, we chose allogenic bone marrow-derived MSCs as our positive control—given similar expected effects and based on the experience of our group with bone marrow-derived MSCs. In the second phase of this study, we report that there were no significant differences in treatment effects of intra-articular allogenic MSC-secretome and allogenic MSCs in this model of joint inflammation. We consider this to be a positive finding, considering that the allogenic MSCs are now generally accepted to be safe for use in equine joints (45), and safety and efficacy have been further validated by European Medicine Agency authorizations (4). We also observe in our study that a second dose of secretome did not result in increased inflammatory responses when compared to MSCs injection. However, it is challenging to compare our results to other studies investigating the effects of allogenic MSCs in equine joints, given the differences in MSC sources, experimental models, and outcome measures reported. As we did not directly test the efficacy of allogenic MSCs by comparing them to a negative control while we can conclude that in the second phase of our study the efficacy of MSCs and MSC-secretome are equivocal the possibility that neither are effective in this model of inflammation cannot be ruled out. It must be acknowledged that the effect on clinical measurements seen in the first phase of this study while significant is quite small, and it is unclear whether these would translate to clinical benefit. This finding is perhaps disappointing, particularly compared to the more positive results reported by Williams et al. for their umbilical derived MSCs (46). However, there are many differences between the models used, not least the source of MSCs, the dose of LPS and the timing of treatment. We believe that our results do support the overall conclusions from other studies (3, 46, 47), that allogenic MSCs but also allogenic MSC-secretome are safe for use and warrant further investigation.

A significant weakness in this study is the limited characterization of the therapeutic treatments investigated. While we have previously used the techniques described to produce MSC secretome from human MSCs (17), it would have been useful to further characterize the therapeutic produced here from equine MSCs. In the absence of further evaluation of the product, it is difficult to predict what therapeutic effects it could be expected to have, and it is clear that species differences can be expected. For example, in the study by Khatab et al. investigation of human MSC-derived secretome, indoleamine 2,3-dioxygenase (IDO) activity was measured to confirm the anti-inflammatory potential of donors but such assay was not even possible for the equine donors as equine MSCs do not produce IDO (48). Further evaluation of the equine MSC-secretome produced using the described techniques, which at the minimum should involve measurement of some expected inflammatory cytokines in the product should be included in any future studies. Similarly, we would consider it essential in future studies to include further characterization of the MSCs used. While previously published studies and other studies using MSCs isolated and cultured using these methods can give us some insight into the expected traits of these cells for this study (23, 25–28) specific characterization of the pooled MSCs used for the current study was regrettably not performed. Future work should include at least the suggested minimal definitions for equine MSCs as set out in a recent position study (24). This would not only allow for better standardization of the MSCs used and therefore of the secretome obtained, but also allow for easier comparison of these with MSCs and MSC-based products investigated by other research groups. The limited amount of characterization in the current study means that the previously mentioned disappointing comparison with other studies or reported success in clinical cases is perhaps then not surprising, as we cannot be sure that we are comparing similar products.

The horse is a particularly interesting experimental model for joint research, being both a target species for novel therapeutics and a suitable translational model (49, 50). Based on *in vitro* findings regarding differences in the behavior of MSCs in inflammatory environments it appears that testing the safety and potential efficacy of allogeneic MSCs using experimental models of inflammation may be particularly important (45). Previous studies examining the effects of MSCs in an *in vivo* inflammatory joint environment have each used different models of joint inflammation. Williams et al. reported a significant reduction in inflammation when allogenic umbilical cord blood-derived MSCs were administered into joints inflamed with a 0.5 ng dose of LPS (46). Using the more severe amphotericin-B model of joint inflammation, to examine the effects of allogenic bone marrow derived stem cells Barrachina et al. reported that clinical and synovial inflammatory parameters were significantly reduced, and also that the second injection of allogeneic cells yielded no adverse reactions (47). A further study reported by Colbath et al. looking at the effects of allogenic and autogenous bone marrow-derived stem cells in an rIL-1β model of synovial inflammation did not find either type of MSCs to be effective in reducing inflammation (3). While no experimental model will exactly replicate naturally occurring disease, we have chosen to focus on the equine intra-articular LPS synovitis model as our group has extensive experience with this model and it has now been widely used for testing potential therapeutics (31, 32, 34, 35, 46). We have demonstrated that sub nano doses of LPS elicit marked, reliable yet transient effects on certain synovial fluid inflammatory biomarkers, MMP activity, and some markers of cartilage turnover (51). Additionally, synovial fluid biomarkers in horses have been extensively studied (40) and changes in synovial fluid concentrations of the same have been used as outcomes measures in studies investigating the effects of various interventions and therapeutics (35, 52).

One of the main limitations of large animal models, in general, is the inherent variability in biological responses between animals. Within animal controlled models are effective in counteracting this limitation. In addition, bilateral orthopedic models have been proven to significantly enhance statistical power (53). We recently refined our model to ethically allow for animal controlled testing of therapeutics in a bilateral low dose LPS induced inflammation model by using a lower dose of LPS (0.25 ng) (22, 32). A disadvantage of this low dose bilateral model is that it precludes the use of unilateral lameness measurements as an outcomes measure. Lameness assessment is inherently reliant on the ability to detect asymmetry of movement between limbs, which may be absent when bilateral lameness is present (54). We do not believe that any described lameness grading systems are suitable for application to bilateral lameness. Indeed, assigning grades in bilateral lameness is thought by some experts to be potentially misleading (54). Furthermore, while it does produce reliable intra-articular inflammation, it is accepted that doses of <0.5 ng LPS give variable, inconsistent levels of lameness (55). Hence lameness levels in our study, while monitored and recorded as part of the overall composite welfare scores, were not considered to be valid outcome measures in this study and therefore were not evaluated or reported as such.

We believe allogenic MSC-secretome as a treatment of joint inflammation could offer many clinical and logistical advantages over MSCs themselves. The use of allogenic stem cells has previously been acknowledged to have potential medical advantages over autologous cells (2). Allogenic cells may be screened and characterized prior to administration leading to a more consistent, higher quality end product. Ongoing production processes rather than the logistical restraints of multiplying cells from the target animal allow for wider availability and cost effectiveness, which is of particular importance in veterinary medicine (48). In addition to wider accessibility, the off-the-shelf nature of the potential end-product could also allow for more appropriate timing of treatment and repeated treatments where necessary. There is a further potential benefit to MSC-secretome being a cell-free product as it is known that MSCs maintain a certain degree of immunogenicity, particularly after stimulation which is performed to optimize their trophic effects (15, 56, 57). It is expected that the concentration of immune complexes in the secretome is lower than with cells, causing a weaker host inflammatory response (58). MSC-secretome could therefore also be a more attractive product due to the potential risk of immunological reactions to foreign MHC antigens expressed by MSCs (59).

Work outlining the importance of MSC extracellular vesicles and other secreted factors is ongoing (60). As these components become further characterized, we may be better able to direct toward the production of certain trophic factors with the use of specific priming techniques. In addition, optimal dosages and timings need to be determined. In the future we could have the ability to produce more targeted treatments for specific conditions, and stages of the disease. While this study is an important first step to establishing the safety and potential efficacy of MSC-secretome as an intra-articular therapeutic, clearly further investigations are needed. Equally, in the absence of an ideal experimental model for joint inflammation, and as we know different inflammatory environments can stimulate MSCs in different ways, it would be interesting to compare the effects of MSCs and MSC-secretome in different models of intra-articular disease, and even more relevantly in cases of naturally occurring disease.

## Limitations

A number of limitations to this experimental model must be acknowledged. While this low dose intra-articular LPS model certainly produces a reliable intra-articular inflammation, the transient and self-limiting nature of this inflammation is of course not completely reflective of natural disease states, where recurrent episodes of inflammation play a crucial role in development and progression of OA.

A further limitation is that only markers of cartilage metabolism were investigated, and the cartilage in these joints was not directly examined either before (by means of direct arthroscopic visualization and/or biopsy) or after (arthroscopic visualization or post mortem examination) the experimental treatments were administered. It would have been interesting to compare the findings in our biomarkers to any changes in the structure of the cartilage or synovium. Histopathological evaluation of the cartilage for example have helped elucidate the reasons for the differences in GAG levels between treatment groups. However, such examinations were outside of the scope of this study.

The use of the same joints for both phases of the study could also be considered a limitation. Based on our previous work examining the effects of LPS induction and repeated inductions of LPS (22, 51) we know that outcomes measures return to baseline values around 7 days post LPS induction. Therefore, we were confident that leaving 14 days between LPS inductions would be a sufficient period. The return to within or close to baseline ranges seen for the majority of biomarkers by timepoint 168 in Phase 1 would appear to support this. While the minimal effects seen in Phase 1 of the study suggest it is unlikely that there are sustained effects in this joint as we do not know what the duration of effect (if any) of MSC-secretome is, we cannot fully exclude the possibility that in Phase 2 we are seeing the cumulative effect of two doses of MSC-secretome. An Advantage from a safety point of view was that this approach provided the opportunity to evaluate a repeated dose of the MSC-secretome, to assess if there was any obvious evidence of sensitization.

A further limitation to consider with this model is that we have not isolated the potential inflammatory effect of repeated arthrocentesis, which has been previously reported (52, 61). Therefore, it is not possible to determine to what degree the physical insults of arthrocentesis and fluid aspiration may be contributing to the articular inflammatory reaction described, and how much of the reaction is a response to the LPS itself. While this was not addressed here, an earlier study where responses in saline injected control joints were studied showed that while increases in gross markers of inflammation such as total protein and white blood cell counts were seen in control joints (62), these responses were substantially less than the increases noted here. Further studies comparing the effects of absolute controls (saline) to the effects of LPS found that there were substantially greater responses in the LPS injected joints across a range of markers such as prostaglandin E2 and tumor necrosis factor-α (63, 64). Given this evidence and considering the principles of 3 R, we believe that using more animals as controls was not justified, particularly as in this bilateral model each joint undergoes the same degree of “insult” or inflammation induced from the LPS plus the physical effects of sampling across the same timeline and therefore it is the effects of the therapeutics being investigated on the sum of this inflammation that is of interest.

## Conclusions

In conclusion, we have found indications for a small beneficial effect of allogenic MSC-secretome on clinically assessed inflammation as well as an effect on matrix turnover dynamics evaluated by biological markers. Additionally, while further investigations comparing the two both to each other and to negative controls are clearly needed our findings suggest that the treatment effects of allogenic MSC-secretome in this model are comparable to those of intra-articular allogenic MSCs. These results encourage further development of secretome-based strategies for therapeutic use as a durable and off-the-shelf disease modifying anti-osteoarthritic drug.

## Data Availability Statement

The original contributions presented in the study are included in the article/Supplementary Material, further inquiries can be directed to the corresponding author.

## Ethics Statement

The animal study was reviewed and approved by University College Dublin Animal Research Ethics Subcommittee University College Dublin Ireland and Irish Health Products Regulatory Authority and Institutional Animal Care and Use Committee of Colorado State University (harvesting of donor cells).

## Author Contributions

CK participated in the study design, carried out the experimental procedures, performed the statistical analysis, and drafted the manuscript. SK prepared the MSC-secretome and MSCs. JK and LG harvested, characterized, and cultured the MSCs from donor animals. GB drafted the manuscript. NK and SP provided technical support with the synovial fluid analyses, performed the mediator and marker assays, and assisted in manuscript preparation. ML assisted with the experimental procedures, provided technical support with the synovial fluid processing and analyses, and assisted in manuscript preparation. PB, GO, and PV conceived of the study, participated in its design and coordination, and helped draft the manuscript. All authors read and approved the final manuscript.

## Funding

The authors would like to indicate that the data presented in this manuscript are part of a larger study investigating the effects of several intra-articular therapeutics on synovial fluid inflammatory parameters and cartilage biomarkers in inflamed equine joints. That study was partly funded by the UCD Wellcome Institutional Strategic Support Fund Clinical Primer Scheme, UCD Foundation (R14799), Dutch Arthritis Association (LLP-22), and the Dutch Organization for Science, Division Applied and Engineering Sciences (Grant No. 12898).

## Conflict of Interest

The authors declare that the research was conducted in the absence of any commercial or financial relationships that could be construed as a potential conflict of interest.

## Publisher's Note

All claims expressed in this article are solely those of the authors and do not necessarily represent those of their affiliated organizations, or those of the publisher, the editors and the reviewers. Any product that may be evaluated in this article, or claim that may be made by its manufacturer, is not guaranteed or endorsed by the publisher.
