# Supplementary material for: Treatment Effects of Intra-Articular Allogenic Mesenchymal Stem Cell Secretome in an Equine Model of Joint Inflammation
Source: Front Vet Sci. 2022 Jun 22;9:907616. doi: 10.3389/fvets.2022.907616 (PMC9257274; doi:10.3389/fvets.2022.907616)
Supplement: Supplementary file 1 [file Data_Sheet_1.docx]

**Supplemental Information**

**Table S1:** Composite Welfare Score Sheet for the Equine LPS Model

| **Parameter** | **Animal ID** | **Score** | **Date/Time** |
| --- | --- | --- | --- |
| Food and water intake | Normal | 0 |  |
|  | Moderate | 1 |  |
|  | Low | 2 |  |
|  | No food or water intake | 4 |  |
| Clinical parameters | Normal temperature (T), cardiac (C) and respiratory (R)rates | 0 |  |
|  | Slight changes | 1 |  |
|  | T ± 1°C, C/R rates increase more than 30 % | 2 |  |
|  | T ± 2°C, C/R rates increase more than 50 % | 4 |  |
| Natural behaviour | Normal | 0 |  |
|  | Minor changes in behaviour including mobility - increase in lameness | 1 |  |
|  | Less mobile and alert | 2 |  |
|  | Restless or still | 4 |  |
| Provoked behaviour | Normal | 0 |  |
|  | Minor depression or exaggerated response | 1 |  |
|  | Moderate change in expected behaviour | 2 |  |
|  | Reacts violently, or very weak | 4 |  |
|  | **Total** | **0-16** |  |

| **Score** | **Action** |
| --- | --- |
| 0-3 | Normal, no action to be taken |
| 4-8 | Monitor carefully, consider analgesics |
| 9-12 | Seek second opinion from named animal care and welfare officer and/or named veterinary surgeon. Consider euthanasia. |
| 13-16 | Indicates severe pain. Seek immediate second opinion from named veterinary surgeon. Animal withdrawn from project. Based on advice from named veterinary surgeon, initiate appropriate treatment and analgesia. If animal’s symptoms cannot be alleviated, again in consultation with the named veterinary surgeon, consider euthanasia. |

**Table S2 Composite Welfare Scores**

|  | **Timepoint** | | | | | | | | | | | | |
| --- | --- | --- | --- | --- | --- | --- | --- | --- | --- | --- | --- | --- | --- |
| **Phase** | **0** | **2** | **4** | **6** | **8** | **24** | **48** | **72** | **96** | **120** | **144** | **168** |  |
| 1 (MSC- secretome vs Medium) | 0 | 0 | 0 | 4 | 2 | 0 | 0 | 0 | 0 | 0 | 0 | 0 |  |
| 2 (MSC-secretome vs MSCs) | 0 | 0 | 0 | 2 | 2 | 0 | 0 | 0 | 0 | 0 | 0 | 0 |  |

**Table S2 Composite Welfare Score:** This score is a total of scores for each of the following categories: food and water intake; clinical parameters; natural behaviour; and provoked behaviour, over time following induction of inflammation with intra-articular injection of 0.25ng of LPS in the radiocarpal joints of horses at PIH 0. Each of the categories is scored from 0-4, so the total range of scores is 0-16. In Phase 1 joints were treated with either MSC-secretome or a similar volume of medium (control) at PIH 2 and in Phase 2 joints were treated with either MSC-secretome or MSCs at PIH 2. Data correspond to the mode (n = 8 joints for each treatment group).

**Table S3 Clinical Parameters MSC Secretome vs Medium (control)**

|  | **Treatment** | **Timepoint** | | | | | | | | | | | |
| --- | --- | --- | --- | --- | --- | --- | --- | --- | --- | --- | --- | --- | --- |
|  |  | **0** | **2** | **4** | **6** | **8** | **24** | **48** | **72** | **96** | **120** | **144** | **168** |
| **Joint Effusion Score** | MSC-secretome | 0 | 0 | 1 | 1 | 1 | 2 | 2 | 1 | 1 | 0 | 0 | 0 |
|  | Medium (control) | 0 | 0 | 1 | 2 | 1 | 3 | 2 | 1 | 1 | 0 | 0 | 0 |
| **Joint Circumference (cm)** | MSC-secretome | 28.81 ± 0.98 | 28.94 ± 0.99 | 29.15 ± 0.99 | 29.48 ± 1.09 | 29.58 ± 1.10 | 30.84 ± 1.18 | 30.63 ± 1.05 | 30.29 ± 1.12 | 29.83 ± 1.13 | 31.30 ± 1.21 | 29.54 ± 1.20 | 29.35 ± 1.17 |
|  | Medium (control) | 28.75 ± 0.82 | 28.85 ± 0.83 | 29.05 ± 0.97 | 29.49 ± 1.07 | 29.61 ± 1.09 | 31.15 ± 1.27 | 30.91 ± 1.23 | 30.65 ± 1.17 | 30.34 ± 1.22 | 30.09 ± 1.17 | 29.98 ± 1.22 | 29.80 ± 1.18 |

**Table S3: Clinical Parameters MSC Secretome vs Medium (control)**

*Joint Effusion* is a score (scale 0-4) for observed/palpated joint effusion recorded over time following induction of inflammation with intra-articular injection of 0.25ng of LPS in the radiocarpal joints of horses at PIH 0. Joints were treated with either MSC-secretome or a similar volume of medium (control) at PIH 2. n = 8 joints for each treatment group. Data correspond to the mode.

*Joint Circumference* is the joint circumference measurement at each time-point following induction of inflammation with intra-articular injection of 0.25ng of LPS in the radiocarpal joints of horses at PIH 0. Joints were treated with either MSC-secretome or a similar volume of medium (control) at PIH 2. n = 8 joints for each treatment group. Data correspond to the mean ± standard deviation of the mean.

**Table S4 Synovial Fluid Analysis MSC Secretome vs Medium (negative control)**

|  | **Treatment** | **Timepoint** | | | | | **Lab Baseline Range** |
| --- | --- | --- | --- | --- | --- | --- | --- |
|  |  | **0** | **8** | **24** | **72** | **168** |  |
| **Total Protein**  **(g/L)** | MSC-secretome | 13.13 ± 5.49 | 59.75 ± 4.95 | 60.25 ± 4.83 | 41 ± 10.02 | 31.75 ± 3.62 | < 20 |
|  | Medium (control) | 11.25 ± 3.54 | 57.75 ± 4.46 | 57.5 ± 8.47 | 41.5 ± 7.84 | 33.25 ± 3.37 |  |
| **WBCC**  **Cells x 10^9^/L** | MSC-secretome | 0.02 ± 0.03 | 253.31 ± 177.64 | 124.60 ± 84.10 | 17.88 ± 9.85 | 0.79 ± 0.77 | 0 - 2 |
|  | Medium (control) | 0.00 ± 0.01 | 212.23 ± 113.53 | 122.19 ± 77.83 | 17.88 ± 14.53 | 0.69 ± 0.45 |  |
| **PGF2α (pg/mL)** | MSC-secretome | 17.33 ± 4.36 | 2203.08 ± 2000.12 | 91.50 ± 61.72 | 36.33 ± 13.37 | 43.64 ± 25.03 | 20 - 100 |
|  | Medium (control) | 20.64 ± 6.99 | 1994.70 ± 1625.36 | 108.19 ± 66.95 | 39.99 ± 18.27 | 53.76 ± 51.53 |  |
| **PGE_2_**  **(pg/mL)** | MSC-secretome | 21.01 ± 6.42 | 13872.95 ± 13725.05 | 602 ± 470.77 | 91.93 ± 42.86 | 56.01 ± 21.66 | 20 - 100 |
|  | Medium (control) | 22.55 ± 6.07 | 10744.44 ± 10225.28 | 921.89 ± 1023.54 | 101.75 ± 53.69 | 168.36 ± 331.17 |  |
| **PGE_1_**  **(pg/mL)** | MSC-secretome | 179.53 ± 101.9 | 10246.5 ± 6788.65 | 712.18 ± 403.4 | 198.21 ± 78.72 | 270.99 ± 56.62 |  |
|  | Medium (control) | 206.43 ± 108.71 | 10538.5 ± 7401.15 | 844.12 ± 636.73 | 337.78 ± 110.48 | 556.92 ± 781.05 |  |
| **LTB_4_**  **(pg/mL)** | MSC-secretome | 16.93 ± 7.75 | 61 ± 29.96 | 70.28 ± 31.09 | 53.77 ± 31.52 | 45.62 ± 29.73 |  |
|  | Medium (control) | 27.38 ± 24.02 | 83.68 ± 63 | 63.2 ± 39.69 | 76.29 ± 78.64 | 57.94 ± 58.81 |  |
| **11-HETE**  **(pg/mL)** | MSC-secretome | 121.15 ± 51.51 | 914.28 ± 1053.64 | 250.1 ± 205.54 | 124.71 ± 53.84 | 149.43 ± 64.05 |  |
|  | Medium (control) | 113.76 ± 57.93 | 546.46 ± 300.14 | 166.23 ± 53.06 | 144.89 ± 68.14 | 121.53 ± 69.85 |  |
| **CCL2 (pg/mL)** | MSC-secretome | 102.13 ± 116.77 | 71368.13 ± 97017.17 | 702.63 ± 458.69 | 238.63 ± 203.88 | 167.63 ± 190.44 | 50 – 100* |
|  | Medium (control) | 111.38 ± 104.82 | 43989.38 ± 42342.12 | 651.50 ± 683.74 | 235.00 ± 185.23 | 252.38 ± 259.89 |  |
| **TNF-α (pg/mL)** | MSC-secretome | 0.00 ± 0.00 | 273.21 ± 191.60 | 55.44 ± 66.07 | 22.58 ± 25.15 | 17.78 ± 31.38 | 0 |
|  | Medium (control) | 0.28 ± 0.78 | 341.53 ± 223.52 | 36.15 ± 49.87 | 24.16 ± 31.03 | 22.29 ± 32.24 |  |
| **MMP (RFU/s)** | MSC-secretome | 29.98 ± 11.48 | 121.94 ± 89.45 | 114.95 ± 47.49 | 141.79 ± 29.87 | 95.21 ± 39.90 | approx. 50 |
|  | Medium (control) | 23.95 ± 12.65 | 129.51 ± 89.13 | 140.56 ± 61.25 | 146.44 ± 53.84 | 116.66 ± 40.97 |  |
| **GAG (µ/mL)** | MSC-secretome | 285.94 ± 110.53 | 149.68 ± 65.83 | 577.34 ± 283.50 | 375.73 ± 95.94 | 117.46 ± 29.89 | 50 - 300 |
|  | Medium (control) | 269.61 ± 82.98 | 120.73 ± 67.70 | 376.05± 174.10 | 340.45 ± 132.58 | 106.44 ± 46.22 |  |
| **C2C (ng/mL)** | MSC-secretome | 233.19 ± 96.08 | 219.44 ± 45.81 | 423.25 ± 187.34 | 372.93 ± 162.88 | 367.19 ± 114.00 | 250 - 350 |
|  | Medium (control) | 202.91 ± 54.45 | 210.94 ± 32.53 | 466.36 ± 166.72 | 384.16 ± 109.22 | 323.38 ± 160.81 |  |
| **CPII**  **(ng/mL)** | MSC-secretome | 2094.57 ± 1939.07 | 2040.35 ± 1112.75 | 3457.83 ± 3721.16 | 3830.93 ± 4319.92 | 3152.11 ± 2472.29 | approx. 1000  * |
|  | Medium (control) | 1653.74 ± 888.17 | 1888.55 ± 485.17 | 2078.10 ± 2313.17 | 3744.100 ± 4516.21 | 2787.14 ± 3298.96 |  |

**Table S4: Synovial Fluid Analysis** **MSC Secretome vs Medium (negative control)**

Comparison of Synovial Fluid Total Protein (TP), White Blood Cell Count (WBCC), Prostaglandin F2α (PGF2α), Prostaglandin E_2_ (PGE_2_), Prostaglandin E_1_ (PGE_1_), Leukotriene B_4_ (LTB_4)_ and 11-hydroxyeicosatetraenoic acid (11-HETE)_,_ CCL2, Tumour necrosis factor-α (TNF-α), general matrix metalloproteinase activity (MMP), Glycosaminoglycan (GAG), collagen-cleavage neoepitope of type II collagen (C2C) and carboxypropeptide of type II collagen epitope (CPII) over time following induction of inflammation with intra-articular injection of 0.25ng of LPS in the radiocarpal joints of horses at PIH 0. Joints were treated with either MSC-secretome or a similar volume of medium (control) at PIH 2. n = 8 joints for each treatment group. Data correspond to the mean ± standard deviation of the mean. In the final column are the expected baseline ranges for our laboratory based on other studies by our group. * denotes assays where we see tend to see particularly large variations in baseline values.

**Table S5 Clinical Parameters MSC Secretome vs MSCs (positive control)**

|  | **Treatment** | **Timepoint** | | | | | | | | | | | | |
| --- | --- | --- | --- | --- | --- | --- | --- | --- | --- | --- | --- | --- | --- | --- |
|  |  | **0** | **2** | **4** | **6** | **8** | **24** | **48** | **72** | **96** | **120** | **144** | **168** |  |
| **Joint Effusion Score** | MSC-secretome | 0 | 0 | 1 | 1 | 1 | 2 | 1 | 0 | 0 | 0 | 0 | 0 |  |
|  | MSCs | 0 | 0 | 1 | 1 | 1 | 1 | 1 | 0 | 0 | 0 | 0 | 0 |  |
| **Joint Circumference (cm)** | MSC-secretome | 29.80 ± 1.15 | 29.85 ± 1.16 | 30.10 ± 1.05 | 30.46 ± 1.05 | 30.66 ± 1.12 | 31.26 ± 0.96 | 30.78 ± 1.13 | 30.36 ± 1.18 | 30.04 ± 1.13 | 29.76 ± 1.17 | 29.85 ± 1.16 | 29.73 ± 1.07 |  |
|  | MSCs | 29.93 ± 1.21 | 29.98 ± 1.21 | 30.21 ± 1.18 | 30.61 ± 1.27 | 30.91 ± 1.13 | 31.58 ± 1.12 | 31.13 ± 1.27 | 30.58 ± 1.23 | 30.36 ± 1.16 | 30.26 ± 1.11 | 30.18 ± 1.21 | 30.05 ± 1.21 |  |

**Table S5: Clinical Parameters**

*Joint Effusion* is a score (scale 0-4) for observed/palpated joint effusion recorded over time following induction of inflammation with intra-articular injection of 0.25ng of LPS in the radiocarpal joints of horses at PIH 0. n = 8 joints for each treatment group. Data correspond to the mode.

*Joint Circumference* is expressed the joint circumference measurement at each time-point following induction of inflammation with intra-articular injection of 0.25ng of LPS in the radiocarpal joints of horses at PIH 0. Joints were treated with either MSC secretome or MSCs at PIH 2. n = 8 joints for each treatment group. Data correspond to the mean ± standard deviation of the mean.

T**able S6 Synovial Fluid Analysis MSC Secretome vs MSCs (positive control)**

|  | **Treatment** | **Timepoint** | | | | | **Lab Baseline Range** |
| --- | --- | --- | --- | --- | --- | --- | --- |
|  |  | **0** | **8** | **24** | **72** | **168** |  |
| **Total Protein**  **(g/L)** | MSC-secretome | 21.71 ± 4.07 | 59.75 ± 4.46 | 55.50 ± 3.66 | 36.25 ± 7.67 | 27.75 ± 9.35 | < 20 |
|  | MSCs | 20.50 ± 3.51 | 59.75 ± 3.66 | 51 ±6.59 | 38.5 ± 4.50 | 32.25 ± 8.71 |  |
| **WBCC**  **Cells x 10^9^/L** | MSC-secretome | 0.33 ± 0.20 | 171.39 ± 48.53 | 95.16 ± 23.28 | 8.77 ± 8.79 | 0.31 ± 0.31 | 0 - 2 |
|  | MSCs | 0.24 ± 0.11 | 174.73 ± 55.31 | 69.57 ± 34.20 | 7.94 ± 7.70 | 0.44 ± 0.52 |  |
| **PGF2α (pg/mL)** | MSC-secretome | 18.36 ± 4.63 | 1854.66± 1403.21 | 136.01 ± 117.00 | 42.58 ± 15.06 | 38.24 ± 13.53 | 20 - 100 |
|  | MSCs | 18.65 ± 5.26 | 2285.08 ± 1867.87 | 146.10 ± 167.22 | 50.79 ± 24.13 | 37.16 ± 7.91 |  |
| **PGE_2_**  **(pg/mL)** | MSC-secretome | 22.74 ± 5.98 | 17855.36 ± 14443.44 | 1072.33 ± 714.85 | 98.24 ± 28.84 | 74.94 ± 60.38 | 20 - 100 |
|  | MSCs | 23.41 ± 4.45 | 12028.09 ± 3779.02 | 1117.37 ± 1027.89 | 148.41 ± 99.71 | 80.58 ± 51.65 |  |
| **PGE_1_**  **(pg/mL)** | MSC-secretome | 152.4 ± 41.18 | 8908.47 ± 7085.39 | 821.5 ± 492.9 | 289.35 ± 94.17 | 280.84 ± 54.57 |  |
|  | MSCs | 165.79 ± 55.8 | 10860.7 ± 7321.25 | 836.07 ± 632.13 | 350.13 ± 158.13 | 306.31 ± 64.07 |  |
| **LTB_4_**  **(pg/mL)** | MSC-secretome | 28 ± 23.59 | 92.51 ± 39.1 | 70.14 ± 47.93 | 81.85 ± 71.59 | 58.13 ± 41.37 |  |
|  | MSCs | 27.6 ± 14.52 | 86.23 ± 36.59 | 67.57 ± 12.45 | 87.31 ± 61.64 | 41.31 ± 15.27 |  |
| **11-HETE**  **(pg/mL)** | MSC-secretome | 91.3 ± 51.5 | 530.44 ± 723.12 | 181.71 ± 56.43 | 98.87 ± 35.31 | 121.08 ± 60.06 |  |
|  | MSCs | 134.27 ± 35.59 | 617.06 ± 471.63 | 172.77 ± 115.87 | 127.75 ± 150.27 | 133.26 ± 53.12 |  |
| **CCL2 (pg/mL)** | MSC-secretome | 163.86 ± 153.91 | 3966.25 ± 2699.64 | 219.88 ± 228.39 | 217.63 ± 136.48 | 241.00 ± 187.18 | 50 – 100 * |
|  | MSCs | 197.00 ± 135.12 | 4472.14 ± 3619.39 | 282.75 ± 362.16 | 261.50 ± 253.18 | 303.63 ± 227.81 |  |
| **TNF-α (pg/mL)** | MSC-secretome | 5.41 ± 7.18 | 136.28 ± 148.52 | 9.31 ± 17.33 | 10.39 ± 14.32 | 16.06 ± 24.12 | 0 |
|  | MSCs | 1.69 ± 4.77 | 166.73 ± 209.69 | 13.30 ± 23.10 | 16.53 ± 28.67 | 20.70 ± 126.46 |  |
| **MMP (RFU/s)** | MSC-secretome | 75.90 ± 35.32 | 173.60 ± 61.93 | 153.39 ± 56.12 | 142.03 ± 50.08 | 104.20 ± 41.89 | approx. 50 |
|  | MSCs | 63.63 ± 32.14 | 201.69 ± 54.80 | 144.93 ± 26.63 | 144.18 ± 55.34 | 127.58 ± 61.26 |  |
| **GAG (µ/mL)** | MSC-secretome | 189.91 ± 89.00 | 158.88 ± 77.87 | 252.15 ± 80.14 | 204.16 ± 83.53 | 89.06 ± 44.96 | 50 - 300 |
|  | MSCs | 154.16 ± 68.42 | 182.81 ± 71.64 | 176.34 ± 100.06 | 125.95 ± 94.18 | 67.15 ± 39.31 |  |
| **C2C (ng/mL)** | MSC-secretome | 344.71 ± 92.93 | 254.09 ± 105.92 | 472.66 ± 100.69 | 382.46 ± 115.01 | 369.58 ± 122.76 | 250 - 350 |
|  | MSCs | 307.74 ± 85.91 | 250.60 ± 93.67 | 484.69 ± 187.97 | 454.31± 250.23 | 394.89 ± 106.56 |  |
| **CPII**  **(ng/mL)** | MSC-secretome | 2273.41 ± 1599.60 | 2467.06 ± 1091.01 | 6328.89 ± 3517.10 | 2951.61± 1749.63 | 4892.72 ± 5420.77 | approx.. 1000 * |
|  | MSCs | 3743.85 ± 4276.55 | 2875.11 ± 1829.41 | 4510.74 ± 1866.50 | 2911.80± 2152.44 | 2645.51 ± 1790.07 |  |

**Table S6: Synovial Fluid Analysis** **MSC Secretome vs MSCs (positive control)**

Comparison of Synovial Fluid Total Protein (TP), White Blood Cell Count (WBCC), Prostaglandin F2α (PGF2α), Prostaglandin E_2_ (PGE_2_), Prostaglandin E_1_ (PGE_1_), Leukotriene B_4_ (LTB_4)_ and 11-hydroxyeicosatetraenoic acid (11-HETE), CCL2, Tumour necrosis factor-α (TNF-α), general matrix metalloproteinase activity (MMP), Glycosaminoglycans (GAG), collagen-cleavage neoepitope of type II collagen (C2C) and carboxypropeptide of type II collagen epitope (CPII) over time following induction of inflammation with intra-articular injection of 0.25ng of LPS in the radiocarpal joints of horses at PIH 0. Joints were treated with either MSC-secretome or MSCs at PIH 2. n = 8 joints for each treatment group. Data correspond to the mean ± standard deviation of the mean. In the final column are the expected baseline ranges for our laboratory based on other studies by our group. * denotes assays where we see tend to see particularly large variations in baseline values.
